# Anti-arthritic activity of *Ricinus communis* L. and *Withania somnifera* L. extracts in adjuvant-induced arthritic rats *via* modulating inflammatory mediators and subsiding oxidative stress

**DOI:** 10.22038/ijbms.2021.55145.12355

**Published:** 2021-07

**Authors:** Asif Hussain, Bilal Aslam, Faqir Muhammad, Muhammad Naeem Faisal, Shaneel Kousar, Aqsa Mushtaq, Muhammad Usman Bari

**Affiliations:** 1 Institute of Physiology and Pharmacology, University of Agriculture, Faisalabad-38040, Faisalabad, Punjab, Pakistan

**Keywords:** Anti-oxidants, Inflammatory cytokines, Oxidative stress, Rheumatoid arthritis, Ricinus communis, Withania somnifera

## Abstract

**Objective(s):**

This study aimed to evaluate the anti-arthritic activity of *Ricinus communis* leaves’ and *Withania somnifera* roots’ hydroalcoholic extracts in Complete Freund’s adjuvant-induced arthritis in Wistar rats.

**Materials and Methods:**

HPLC and FT-IR analysis detected pharmacologically important phytocompounds in both plant extracts. Oral treatments including methotrexate (MTX; 3 mg/kg twice a week) and extracts at 250 and 500 mg/kg/day were initiated after arthritis induction. Changes in paw swelling, arthritic score, body weight, organ indices (thymus and spleen), hematological and biochemical parameters, and pro-/anti-inflammatory cytokine expression using qRT-PCR were assessed. Oxidative stress markers in hepatic tissue were determined. Histopathological and radiological examinations were also performed.

**Results:**

RCE (*R. communis* extract) and WSE (*W. somnifera* extract) demonstrated a reduction in paw swelling, arthritic score, and restoration of body weight and organ indices. Hematological parameters, serum inflammatory markers such as CRP and RF, and liver function markers of arthritic rats were significantly (*P*<0.01) ameliorated with RCE and WSE treatment. Both plants persuasively down-regulated IL-1β, IL-6, IL-17a, TNF-α, and RANKL and up-regulated IL-4, INF-γ, and OPG relative expression as well as alleviating hepatic oxidative stress parameters. Histopathological and radiological findings revealed a marked reduction in tissue inflammation and bone erosion in extracts treated groups.

**Conclusion:**

The study findings suggest that *R. communis* leaves and *W. somnifera* roots have markedly subsided inflammation and improved health through modulating pro-/anti-inflammatory cytokine expression and reducing oxidative stress.

## Introduction

Rheumatoid arthritis (RA) is primarily a polyarticular tissue affecting autoimmune and inflammatory disease that mainly affects the peripheral joints, and damages cartilage and bones (1). Nearly 0.5% to 1% of the adult population of the world is suffering from RA and displayed high risk of morbidity and mortality in the last decade. RA is more prevalent in males than in females (2). Despite advances in the scientific research field, the etiology of RA is still unclear. However, pathological features of RA include cartilage and articular damage, progressive synovitis, ankylosis, and severe pain which progress to irreversible joint damage and disability (3). Individual inherited susceptible genes are prone to environmental factors such as dust exposure, cigarette smoking, and infections which trigger these genes, and consequently induce an abnormal immune response that attacks the joints (4). The generation of reactive oxygen species (ROS) from metabolic processes has an important role in RA pathogenesis as they contribute to joint tissue damage (5). Anti-oxidants can scavenge free radicals and reduce tissue damage, however, an imbalance between pro-oxidants and anti-oxidants in arthritis has been assumed due to increased cellular activities and inefficient anti-oxidant defense mechanisms (6).

Studies reported the pivotal role of enhanced expression of inflammatory transcription factors and cytokines that are linked to joint destruction (7). The pathogenesis and progression of RA involved overexpression of pro-inflammatory cytokines, including TNF-α, IL-1β, IL-6, IL-17a, and prostaglandins (PGE_2_) (8, 9). TNF-α accelerates inflammation amplification *via* synovial fibroblast stimulation which up-regulates cellular adhesion of mediators and enhances leukocyte migration leading to joint damage. IL-6 stimulates blood vessel growth and promotes inflammation. IL-1β regulates bone resorption, cartilage damage, and can modify nitric oxide (NO) and PGE_2_ production, while PGE_2_ stimulates fever induction and pain receptors. Furthermore, secretion of IL-17a from T helper (Th) 17 cells amplifies synovial inflammation and damage, often through synergistic interactions with TNF-α and IL-1β, by increasing the relative expression of IL-6 and IL-8 (10). In addition, anti-inflammatory cytokines include IL-4, secreted from activated T lymphocytes, mast cells, and macrophages, which can suppress the relative expression of pro-inflammatory cytokines (TNF-α, IL-1β, IL-6, and PGE_2_) and up-regulate anti-inflammatory mediators such as IL-1 receptor agonists (11). INF-γ ameliorates inflammatory response by inhibiting Th-17 cell differentiation and osteoclasts (12). Thus, synovial membrane inflammation and joint damage can be a consequence of pro-inflammatory and anti-inflammatory state imbalance (9, 13). OPG/RANKL is considered a vital bone tissue metabolism-regulating system and modulates osteoclastogenesis (14).

Currently, RA treatment includes anti-inflammatory drugs, steroid hormones (glucocorticoids), anti-rheumatic drugs (DMARD), biological agents, and immunosuppressants (15). However, these treatments are costly, inconvenient, and their prolonged use at higher doses is associated with adverse effects such as cardiovascular complications, gastrointestinal and hepatic disorders, hormonal imbalance, and immunodeficiency (16). Thus, therapeutic strategies for RA require comparatively safe and economical drugs that can prevent joints damage.

Medicinal plants are getting more attention as a potential source of safe and cost-effective anti-rheumatic agents. Herbs may be used as an effective alternative treatment for inflammatory diseases (17). In folk medicine, leaves of *R. communis *L. (family Euphorbiaceae), famous as ‘castor oil plant’, are traditionally used to treat inflammation (18). *R. communis *is a tropical flowering plant that is widely cultivated in Asian countries. Studies on *R. communis *leaves have shown anti-oxidant, antibacterial, hepatoprotective (18, 19), anti-nociceptive effect (20), osteoarthritic and anti-cancer activities (21). *W. somnifera *L. Dunal (family Solanaceae) is widely distributed in Asian countries (22). *W. somnifera *has been used as a traditional remedy due to its anti-microbial, anti-oxidant, anti-diabetic (23), anti-cancer, chondroprotective, cardioprotective, immunomodulatory, and hepatoprotective properties (24). Based on traditional uses of *R. communis* leaves and *W. somnifera *roots, the present study was carried out to evaluate their ameliorative effects on joint inflammation mediated by pro-/anti-inflammatory cytokines, and alleviation of oxidative stress in adjuvant-induced arthritis in Wistar rats.

## Materials and Methods

***Drugs and chemicals***

Complete Freund’s adjuvant (InvivoGen^®^, France), methotrexate (Tablet Cytotrexate^®^, Lahore Chemical & Pharmaceutical Work Pvt., Ltd., Pakistan), trizol (Trizole reagent; Thermo-Scientific^®^, UK), cDNA synthesis kit (Thermo-Scientific^®^, UK), Maxima Syber Green/ROX Master Mix 2X (Thermo-Scientific^®^, UK), nuclease-free water (Ambion-Thermo-Fisher^®^, USA), Oligo-primers (Macrogen^®^, USA), and methanol (Merck^®^) were purchased.

***Plant collection***

*R. communis* leaves and *W. somnifera* roots were collected in March 2019 from the Botanical garden of UAF, authenticated by a taxonomist, and deposited to the herbarium of the Department of Botany, UAF (herbarium number: *R. communis*; 212-1-19, *W. somnifera*; 212-2-19) for future reference.

***Preparation of extracts***

*R. communis *leaves and *W. somnifera *roots were washed thoroughly and shade dried for 15 days. Then, pulverized and sieved to get a uniform powder. About 200 g of each powdered plant was macerated with 5 L of solvent (water: ethanol, 30:70 v/v) for five days with shaking at regular intervals. Extracts were filtered, lyophilized, and concentrated at 40–60 °C under reduced pressure using a rotary evaporator (Heizbad Hei-VAP, Heidolph^®^, Germany). This process was repeated three times to gain maximum yield. The percentage yields of concentrated extracts were 19.3% and 13.8% (w/w), respectively.

***Phytochemical characterization***

A modified method (25) was used for the quantification of flavonoid and phenolic compounds present in extracts using an HPLC instrument (Shimadzu, Japan) accompanied by a C_18_ column and UV-visible detector (Shimadzu, Japan). The EZchrom elite^®^ software package was used for data acquisition. Briefly, 50 mg of each dried extract was dissolved in 40 mL of methanol (60%), acidified with HCl, and heated for 2 hr at 90 °C. The mobile phase comprised A: 6% (v/v) of acetic acid (pH=2.27) and B: 100% (v/v) of acetonitrile with a gradient elution: 15%, 45%, and 100% for 15 min. A sample volume of 20 µl was injected, maintaining the mobile phase flow rate at 1 mL, column temperature at 27 °C, and absorbance was taken at 280 nm. Phytocompounds were quantified by comparing the concentrations and retention times (26).

FT-IR analysis was performed to detect different types of bonds and functional groups in RCE and WSE. In short, each dried extract weighing 10 mg was used for FT-IR spectroscopy (Spectrum Two^™^, Perkin Elmer) with a scan range of 4000–600 cm^-1^ and 4 cm^-1 ^resolution. A previously described method was adopted for analysis (27).

***Experimental animals***

Forty-two healthy male Wistar rats (age: 6 to 8 weeks, weight range: 150–180 g) were procured and placed at the animal house facility of the Institute of Microbiology, UAF. Prior to conduct trial, animals were grouped on body weight basis and acclimatized for one week, providing standard laboratory conditions, i.e., pellet chow diet twice a day and fresh water *ad libitum*, temperature (25±2 °C), air humidity (50±10%), and 12 hr light/dark cycle throughout the study.

***Ethical approval***

The study protocols were approved by the Ethical Standards of Animal Care and Institutional Bioethical Committee (IBC) of UAF (D. No. 3498/ORIC; Dated: 22-05-2019). All animals were cared for and treated according to the NIH guidelines for the care and use of laboratory animals (NIH Publication No. 85-23, revised 2002).

***Study design***

Adult Wistar rats were divided into seven groups (n=6). On the first day, 100 µl of CFA was injected into the sub-plantar region of the left hind paw to induce arthritis. Treatments were initiated on day 8 by dissolving MTX and extracts in distilled water and administered *via* oral route till 24th day of study. Acute oral toxicity study of *R. communis *leaf aqueous-ethanol extract (28) and *W. somnifera *methanol extract (29) showed a safe profile up to 2 g/kg of body weight, PO in Wistar rats. Therefore, four groups were treated with 250 and 500 mg/kg/day doses of extracts. MTX at 3 mg/kg twice a week dose was selected as used in previous studies (30, 31). Oral treatments were given as:

Group I NC: Normal rats were given 3 ml/kg/day of distilled water

Group II AC: Arthritic rats received 3 ml/kg/day of distilled water

Group III MTX: Arthritic rats received MTX (3 mg/kg twice a week)

Group IV RCE-250: Arthritic rats received RCE (250 mg/kg/day)

Group V RCE-500: Arthritic rats received RCE (500 mg/kg/day)

Group VI WSE-250: Arthritic rats received WSE (250 mg/kg/day) 

Group VII WSE-500: Arthritic rats received WSE (500 mg/kg/day)

***Evaluation of polyarthritis***

The paw diameters were measured using a digital micrometer gauge on day 1 and post-CFA injection effects on paw edema were measured on days 8, 12, 16, 20, and 24. Arthritis severity (arthritic score) was graded in ipsilateral and contralateral paws from to 0 to 4. Grade 0 represents swelling absence; grade 1 indicates mild erythema or inflammation of one of the fingers; grade 2 denotes the swelling of more than one finger; grade 3 shows wrist or ankle inflammation, and grade 4 displays finger or wrist severe arthritic swelling. The highest arthritis score of 8 was fixed for CFA-induced arthritis in rats (32). Pre-/post-CFA injection changes in animal body weight were measured on day 1 and continued at regular intervals (8, 12, 16, 20, and 24 days).

***Blood and organ sampling***

On the 25^th^ day of CFA injection, blood samples were collected in EDTA and gel clot activator tubes through cardiac puncture under the influence of mild anesthesia. After blood collection, all animals were sacrificed by cervical dislocation, and immune organs (thymus and spleen) were collected, and wet organ weight was measured immediately to calculate the immune organ index (organ weight in mg/bodyweight in g) (33). Hind limbs and liver tissues were collected for histopathological examination and paw tissues were immediately stored in RNA ladder for gene expression analysis.

***Determination of hematological and biochemical parameters***

Blood samples stored in EDTA tubes were measured for complete blood count (CBC) using an automated hematology analyzer (Boule Medical AB^®^, SE-12613, Stockholm, Sweden). Serum was separated by centrifuging blood samples at 1010x g for 15 min, and stored at -20 °C in the biomedical freezer (Labfreez^®^ Instruments, China) till further analysis. The levels of C-reactive protein and rheumatoid factor were measured using diagnostic ELISA kits (InvivoGen^®^, Thermo-Fisher). Commercially available kits (QCA^®^, Spain) were used to determine the liver function and renal function biomarkers using an auto-analyzer (Thermo-Scientific Multiskan Go^TM^).

***Gene expression analysis***

Paw tissues were used for assessment of gene expression of IL-1β, IL-4, IL-6, IL-17a, INF-γ, TNF-α, OPG, and RANKL using qRT-PCR analysis (34). TRIzol was used for extraction of mRNA, and yield was determined by a Nanodrop spectrophotometer. The cDNA synthesis kit was used to perform reverse transcription according to the kit manufacturer protocol. Maxima Syber Green/ROX Master Mix 2X, nuclease-free water, and Oligo-primers (provided as **supplementary Table 1**) were used for amplification and quantification. Briefly, 10 µl of Syber mix, 7 µl of nuclease-free water, and 1 µl of each forward and reverse primer were transferred to the 96-wells micro-plate and placed in thermal cycler at 95 °C adjusting 40 cycles of denaturation, and 60 °C for annealing followed by extension at 72 °C. Primer blast and Gene bank^®^ were used for primer designing.

***Preparation of liver tissue homogenate***

The ice-cold 10% solution of potassium chloride was used to prepare 10% w/v of liver tissue homogenate, then centrifuged for 10 min at 3000xg, and divided into various aliquots to use for estimation of catalase (CAT), superoxide dismutase (SOD), and malondialdehyde (MDA) according to standard protocols.

***Determination of oxidative stress biomarkers***

The CAT activity was determined by adding liver tissue homogenate (10% w/v) to H_2_O_2_, and reduction in absorption was measured at 240 nm (35). A change of 0.01 U/min in absorbance was expressed as one unit of CAT, and results were expressed in U/mg protein. The SOD activity in liver homogenate (10% w/v) was determined by its ability to inhibit pyrogallol autoxidation in an alkaline medium, and absorbance was taken at 420 nm. One unit of SOD was considered as a concentration of enzyme that causes 50% inhibition, and estimated results were presented as U/mg protein (36). The thiobarbituric acid reacting substances (TBARS) method was applied to determine the MDA level (37). The absorbance of the reaction mixture containing liver homogenate and reagents was taken at 532 nm, and the MDA level was presented as µM/mg protein.

***Histopathological examination of ankle joints and liver***

Ankle joints and liver tissues were removed, washed immediately with saline, and preserved in a 10% neutral formalin buffered solution for histopathological study. Tissues were processed and embedded in paraffin. After sectioning of tissues (5 mm of thickness), Hematoxylin and Eosin staining of ankle joint and liver sections were done and examined under a light microscope (Olympus PM-10ADS, Olympus optical Co., Tokyo, Japan) using a software package (TOUPCAM^®^, ToupTek Photonics Co., Ltd., China) for the histopathological changes.

***Radiological examination of ankle joints***

Hind limbs of rats were radiologically examined using X-ray unit underexposure of 50 KVp and 200 mAs (*KXO-12R, Toshiba, Japan*). Qualitative assessment was performed for narrowing of joint space, swelling of soft tissues, ankylosed joints, periosteal reaction at the metatarsal area, and osteolysis (38).

***Statistical analysis***

All collected data were statistically analyzed and interpreted by one-way and two-way ANOVA followed by *post-hoc* test, Tukey’s test using GraphPad Prism^®^ version 6.01 (GraphPad, Software Inc., USA), and results presented as mean±SD (n=6). Level of significance *P*<0.05 was used to find differences between controls and extract-treated groups.

## Results

***HPLC and FT-IR analysis***

Quantitative analysis studies showed the presence of gallic acid, quercetin, benzoic acid, caffeic acid, vanillic acid, syringic acid, ferulic acid, and cinnamic acid in RCE while WSE contained gallic acid, quercetin, benzoic acid, caffeic acid, vanillic acid, chlorogenic acid, *m*-coumaric acid,* p*-coumaric acid, and cinnamic acid as shown in Table 1. The highest quantity of gallic acid were found in RCE and of quercetin in WSE. 

The FT-IR results of RCE and WSE showed the presence of flavonoids, amino acids, carbohydrates, primary, secondary, and tertiary alcohols, and aromatic functional groups (39) as summarized in Table 2. Original source data underlying Tables 1 and 2 were provided as supplementary material.

***Effect of RCE/WSE on physical parameters***

**Figure 1:**
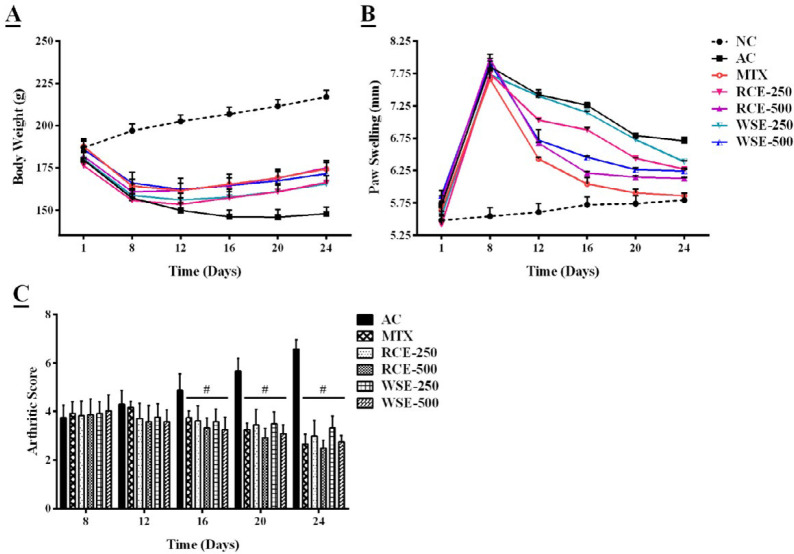
Treatment effects on (A) body weight, (B) paw swelling, and (C) arthritic score of arthritic rats. Results were analyzed by two-way ANOVA, following post-hoc Tukey’s test, and presented as mean±SD (n=6). Significant difference from AC (#*P*<0.05) and MTX (ns *P*>0.05). NC: normal control; AC: arthritic control; MTX: methotrexate; RCE: *Ricinus communis* extract; WSE: *Withania somnifera* extract

The arthritic control group showed a gradual reduction in bodyweight with development of inflammation. Bodyweight loss was significant (*P*<0.01) in arthritic rats, in comparison with normal rats, while MTX, RCE, and WSE treated groups showed significant (*P*<0.05) improvement in body weight of arthritic rats, in comparison with arthritic control from 12^th^ day to the end of the study (Figure 1A).

Post-CFA injection in the sub-plantar surface increased paw swelling, and peak inflammation was observed on the 8^th^ day. Paw swelling was evaluated in arthritic control (*P*<0.01), as compared with normal control from day 8 to 24. Administration of MTX, RCE, and WSE significantly (*P*<0.05) alleviated paw swelling from day 12 onwards, in contrast to arthritic rats. RCE and WSE showed a non-significant (*P*>0.05) difference in decreasing paw swelling at 500 mg/kg, in comparison with MTX treated group (Figure 1B). 

A gradual increase (*P*<0.01) in arthritic scores was observed in arthritic rats (Figure 1C). Experimental groups treated with MTX, RCE, and WSE revealed a marked reduction in arthritic score, in comparison with arthritic control from day 12^th^ to the end of the study. 

***Effect of RCE/WSE on organ indices***

**Figure 2:**
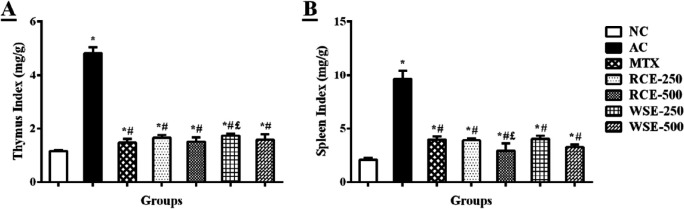
Treatment effects on (A) thymus and (B) spleen indices of arthritic rats. Results were analyzed by one-way ANOVA, following post-hoc Tukey’s test and presented as mean±SD (n=6). *P<0.01, #*P*<0.05, and £*P*<0.05 difference from NC, AC, and MTX, respectively

NC: normal control; AC: arthritic control; MTX: methotrexate; RCE: *Ricinus communis* extract; WSE: *Withania somnifera* extract

Results illustrated in Figure 2(A, B) showed that thymus and spleen weight index significantly (*P*<0.01) increased in arthritic control, in comparison with normal rats. Restoration of thymus and spleen weight index was observed in extracts and MTX treated arthritic rats, in comparison with arthritic control. Maximum effect was noticed at 500 mg/kg dose of both extracts. However, RCE showed more prominent effects than WSE.

***Effect of RCE/WSE on hematological parameters***

Hematological parameters including RBCs, hemoglobin (Hb), WBCs, platelets, hematocrit (Hct), and ESR were estimated in all experimental groups (Table 3). A significant (*P*<0.01) decrease in the levels of RBCs, Hb, and Hct, as well as elevation in the level of platelets, WBCs count, and ESR, were noticed in arthritic control in comparison with normal rats. Treatment with RCE and WSE significantly (*P*<0.05) ameliorated the hematological parameters, as compared with arthritic control.

***Effect of RCE/WSE on serum inflammatory, hepatic, and renal function parameters***

The CFA-induced arthritic control exhibited a significant (*P*<0.01) elevation in serum levels of inflammatory biomarkers including CRP and RF, and liver function biomarkers such as ALP, ALT, and AST, in contrast to normal rats. MTX, RCE, and WSE depicted an ameliorating effect on inflammatory and hepatic function biomarkers. However, both extracts showed a significant (*P*<0.05) effect at 500 mg/kg dose, as compared with arthritic control. A non-significant (*P>*0.05) effect was observed in arthritic control, extracts, and MTX treatments on the level of creatinine and blood urea (Table 4).

***Effect of RCE/WSE on gene expression in paw tissue***

**Figure 3:**
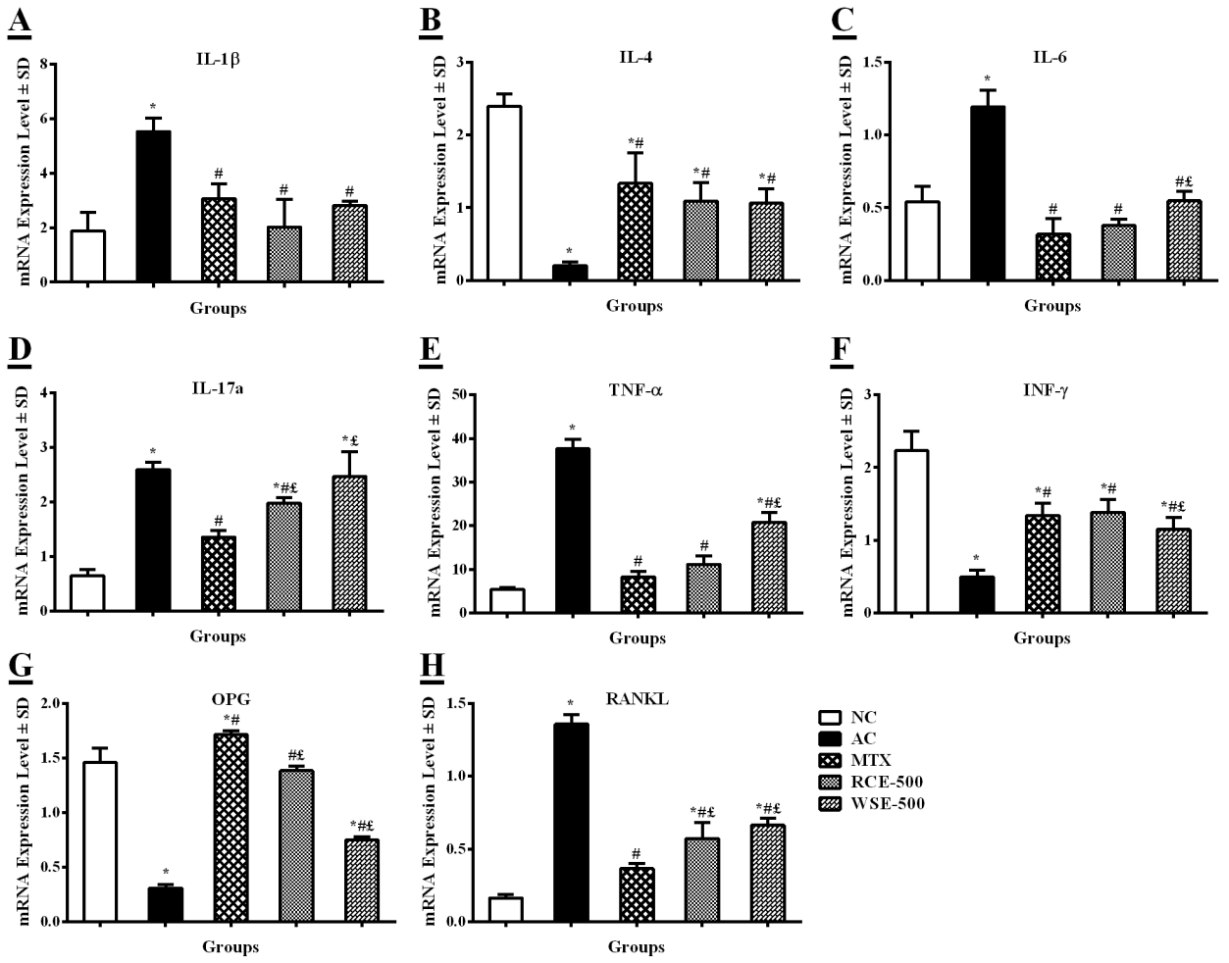
Treatment effects on mRNA relative expression of pro-/anti-inflammatory mediators in arthritic rats. (A) IL-1β: interleukin-1β, (B) IL-4: interleukin-4, (C) IL-6: interleukin-6, (D) IL-17a: interleukin-17a, (E) TNF-α: tumour necrosis factor-α, (F) INF-γ: interferon-γ, (G) OPG: osteoprotegerin, and (H) RANKL: receptor activator of nuclear factor-κB ligand). Results were analyzed by one-way ANOVA, following *post-hoc *Tukey’s test, and presented as mean±SD (n=6). *P<0.01, #*P*<0.05, and £P<0.05 difference from NC, AC, and MTX, respectively. NC: normal control; AC: arthritic control; MTX: methotrexate; RCE:* Ricinus communis* extract; WSE: *Withania somnifera* extract

mRNA expression of pro-/anti-inflammatory cytokines in the paw tissue of experimental arthritic rats was observed at the end of the study (Figure 3). A significant (*P*<0.01) elevation in IL-1β relative expression was observed in arthritic control (5.518±0.498-fold), in contrast to normal rats (1.873±0.683-fold). Arthritic rats treated with MTX (3.067±0.535-fold), RCE at 500 mg/kg (2.020±1.003-fold), and WSE at 500 mg/kg (2.813±0.157-fold) reduced IL-1β relative expression, in comparison with arthritic rats (Figure 3A). A notable (*P*<0.01) reduction in IL-4 relative expression in arthritic control (0.203±0.055-fold) was evaluated, as compared with normal control (2.390±0.151-fold) as shown in Figure 3B. The reduced IL-4 relative expression was increased by MTX (1.333±0.435-fold), RCE at 500 mg/kg (1.090±0.236-fold), and WSE at 500 mg/kg (1.063±0.197-fold) in arthritic rats. An exaggerated IL-6 relative expression (*P*<0.01) was noticed in arthritic control (1.193±0.110-fold), in comparison with normal control (0.540±0.100-fold). Administration of MTX (0.310±0.100-fold), RCE at 500 mg/kg (0.370±0.040-fold), and WSE at 500 mg/kg (0.543±0.059-fold) suppressed IL-6 relative expression, as compared with arthritic control (Figure 3C). A significantly (*P*<0.01) increased IL-17a relative expression was evidenced in arthritic control (2.593±0.133-fold), as compared with normal control (0.640±0.100-fold) that decreased in arthritic rats treated with MTX (1.363±0.117-fold), RCE at 500 mg/kg (1.977±0.097-fold), and WSE at 500 mg/kg (2.473±0.455-fold), as shown in Figure 3D. A considerable (*P*<0.01) upsurge in TNF-α relative expression was observed in arthritic control (37.607±2.141-fold), in contrast to the normal group (5.420±0.423-fold) that down-regulated in response to MTX (8.327±1.214-fold), RCE at 500 mg/kg (11.177±1.951-fold), and WSE at 500 mg/kg (20.790±2.112-fold) treatment (Figure 3E). The relative expression of INF-γ was notably (*P*<0.01) reduced in arthritic rats (0.490±0.092-fold), in comparison with healthy rats (2.230±0.267-fold) that markedly up-surged with the administration of MTX (1.343±0.161-fold), RCE at 500 mg/kg (1.380±0.177-fold), and WSE at 500 mg/kg (1.157±0.166-fold), as expressed in Figure 3F. As shown in Figure 3G, the relative gene expression of OPG was significantly (*P*<0.01) down-regulated in arthritic control (0.310±0.030-fold), as compared with normal rats (1.457±0.135-fold). Arthritic rats treated with MTX (1.710±0.030-fold), RCE at 500 mg/kg (1.387±0.040-fold), and WSE at 500 mg/kg (0.750±0.030-fold) up-regulated OPG relative expression, in contrast to the arthritic group. The RANKL relative expression was significantly (*P*<0.01) elevated in arthritic control (1.350±0.062-fold), in comparison with normal rats (0.160±0.020-fold). Treatment of arthritic rats with MTX (0.360±0.040-fold), RCE at 500 mg/kg (0.570±0.120-fold), and WSE at 500 mg/kg (0.660±0.053-fold) decreased the mRNA expression of RANKL 

***Effect of RCE/WSE on oxidative stress in liver***

**Figure 4:**
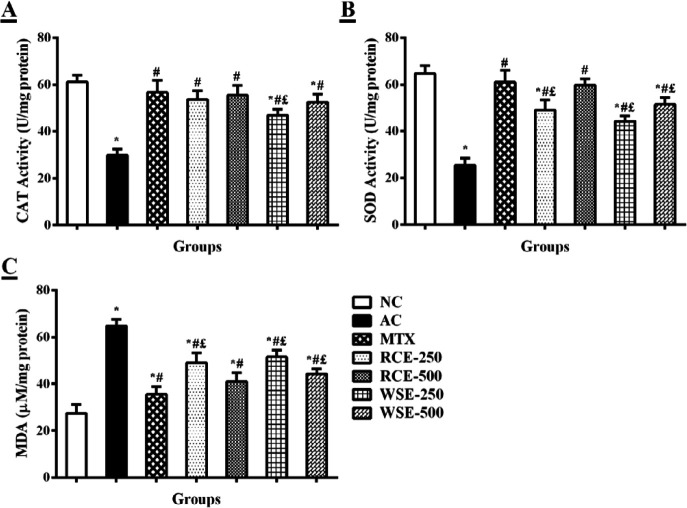
Treatment effects on oxidative stress markers (A) CAT: catalase activity, (B) SOD: superoxide dismutase activity, and (C) MDA: malondialdehyde level in liver tissue. Results were analyzed by one-way ANOVA, following *post-hoc* Tukey’s test, and presented as mean±SD (n=6). *P<0.01, #*P*<0.05, and £P<0.05 difference from NC, AC, and MTX, respectively. NC: normal control; AC: arthritic control; MTX: methotrexate; RCE: *Ricinus communis* extract; WSE: *Withania somnifera* extract

The results of CFA-injection induced oxidative stress in liver tissue, and alleviating effects of MTX, RCE, and WSE are expressed in Figure 4. Test findings showed significant reduction (*P*<0.01) in CAT (29.83±2.64 U/mg protein) and SOD (25.50±3.02 U/mg protein) activities in arthritic control, as compared with normal rats (CAT: 61.17±2.86 U/mg protein and SOD: 64.67±3.39 U/mg protein). Arthritic rats treated with MTX and different doses of extracts markedly (*P*<0.05) improved CAT and SOD activities, as compared with arthritic control (Figures 4A, B). While, RCE at 500 mg/kg produced a non-significant (*P>*0.05) effect, in comparison with MTX. An elevation in serum MDA level of arthritic control (64.67±3.01 µM/mg protein) was evidenced, in contrast to normal control (27.45±3.76 µM/mg protein). Treatment with extracts and MTX significantly (*P*<0.05) decreased MDA level in arthritic rats (Figure 4C). Original source data underlying Figures 1-4 were provided as supplementary material.

***Histopathological examination***

**Figure 5:**
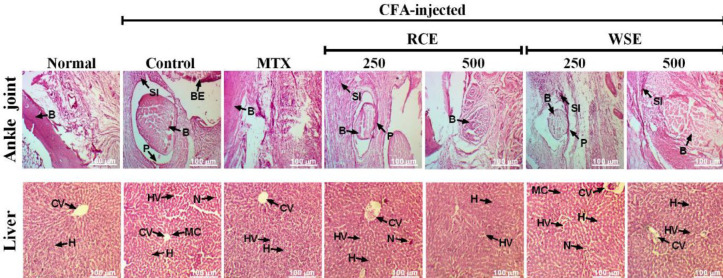
Histopathology of ankle joint of CFA-injected arthritic rats; Normal control showing normal histological features of bone (B) and tissues, Arthritic control indicating bone erosion (BE), pannus formation (P), and synovial infiltration of mononuclear cells (SI), arthritic rats treated with MTX, RCE, and WSE at 500 mg/kg showing less degenerative changes, as compared with rats treated with extracts at 250 mg/kg (Panel-A). Liver tissues indicating normal hepatocytes (H) and central vein (CV), arthritic control exhibiting histopathological changes, i.e., hepatocytes necrosis (N), hepatocytes vacuolization (HV), and mononuclear cell infiltration (MC) in arthritic control, while arthritic rats treated with MTX, RCE, and WSE demonstrated markedly less histopathological alterations (magnification: x100; scale bar: 100 µm)

MTX: methotrexate; RCE: *Ricinus communis* extract; WSE: *Withania somnifera* extract

Histopathology of ankle joint showed marked infiltration of mononuclear cells, bone erosion, and pannus formation in arthritic control, as compared with normal rats (Figure 5; Panel-A). Arthritic rats treated with RCE and WSE revealed a marked reduction in inflammatory cell infiltration, bone erosion, and pannus formation, in comparison with arthritic control. Maximum ameliorative effects were observed at 500 mg/kg of each plant extract comparable with MTX. Histological study of liver evidenced hepatocellular damage by marked vacuolization, congestion of nuclei, and necrosis in arthritic rats, in comparison with normal control (Figure 5; Panel-B). However, treatment with MTX, RCE, and WSE have shown ameliorated effects with significant restoration of hepatic cell vacuolization, nuclei congestion, and necrosis.

***Gross and radiological examination of ankle joint***

**Figure 6:**
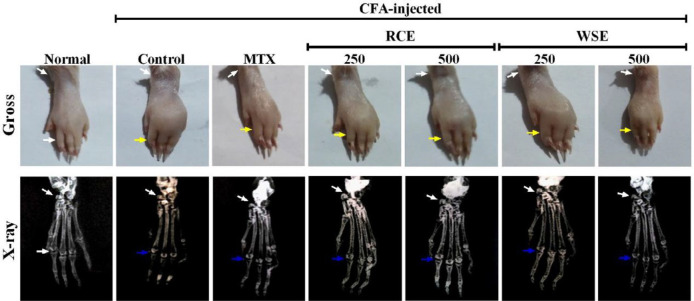
Gross and radiological examination (X-ray) of the left hind paw of CFA-injected arthritic rats. Normal control indicates the absence of degenerative changes. Arthritic control demonstrates ankle joint abrasion (white arrow), inflammation around soft tissues (yellow arrow), and articular damage (blue arrow), while these alterations are markedly decreased in arthritic rats treated with methotrexate (MTX), RCE (250 and 500 mg/kg), and WSE(250 and 500 mg/kg), respectively

MTX: methotrexate; RCE: *Ricinus communis* extract; WSE: *Withania somnifera* extract

Radiological examination of hind limbs of arthritic control exhibited ankylosed joint, soft tissue swelling, osteolysis, narrowing of joint space, and periosteal reaction at a metatarsal area that was in consistency with arthritic score and paw inflammation, as compared with normal rats (Figure 6). MTX-treated arthritic rats markedly reduced joint space narrowing and bone destruction with mild soft tissue inflammation. Treatment with RCE and WSE depicted mild periosteal reaction at the metatarsal area and paw swelling with less joint ankylosis, in comparison with the arthritic group.

**Table 1:** Phytocompounds detected in RCE and WSE by HPLC analysis

Phytocompounds	RCE					WSE			
	Retention time (min)	Area (mV.s)	Area (%)	Conc. (ppm)		Retention time (min)	Area (mV.s)	Area (%)	Conc. (ppm)
**Quercetin**	3.28	2576.23	1.9	136.52		3.10	3076.97	10.8	163.02
**Gallic acid**	4.86	32031.94	10.8	1153.11		4.39	1406.00	4.9	50.61
**Caffeic acid**	12.69	1654.84	1.6	76.18		12.16	281.95	1.0	12.92
**Vanillic acid**	13.54	10611.74	3.6	657.88		13.35	366.07	1.3	22.69
**Benzoic acid**	14.70	5118.79	1.7	542.51		14.64	87.04	0.3	9.22
**Chlorogenic acid**	–	–	–	–		15.99	1204.74	4.2	93.96
**Syringic acid**	16.36	4192.85	1.4	104.81		16.74	335.63	1.2	8.37
***p*** **-Coumaric acid**	–	–	–	–		17.69	1730.93	6.1	22.49
***m*** **-Coumaric acid**	–	–	–	–		20.22	957.61	3.4	11.48
**Ferulic acid**	21.80	12611.59	4.2	907.98		–	–	–	–
**Cinnamic acid**	25.14	18755.07	6.3	376.42		25.21	1199.86	4.2	41.99

RCE: *Ricinus communis* extract; WSE: *Withania somnifera* extract; HPLC: High-performance liquid chromatography

**Table 2:** Functional groups of RCE and WSE detected by FT-IR analysis

RCE			WSE		
**Peak (cm-1)**	Type of bond	Functional groups	Peak (cm-1)	Type of bond	Functional groups
**1710.61**	C=O str.	Flavonoids and lipids	3309.14	O–H str., asymmetric CH2 str.	Alcohols, lipids
**1641.23**	C=O, C=C, N-H str.	Flavonoids: caffeic acid derivatives, Lipids, Amino acids: amide or carboxylic acid	2927.55	C–H str., –CH2 or –CH3 group str.	Vibrations of methyl and methoxy groups in carboxylic acid
**1455.79**	C-H, C=C str., CH2, CH3 str.	Flavonoids and aromatic rings vibrations	1711.26	C=O str.	Carboxyl group, Flavonoids, and Lipids
**1410.71**	C=C str.	Aromatics ring vibration	1515.78	C=C str.	Flavonoids and aromatic rings deformation,
**1264.94**	C–O str.	Polyols such as hydroxyflavonoids	1387.21	CH3(CO) vibration	1,8-Cineole
**1138.15**	C–O-, O–H str.	Tertiary alcohols	1267.96	C–O str.	Polyols such as hydroxyflavonoids
**1046.95**	C-O str., –OH deformation	Tertiary alcohols	1158.04	C–O- str., C–OH bend.	Lipids and alcohol groups
**866.63**	C–O str.	Aromatic ring vibration	1028.04	C–O- str. of the ester group	Primary and secondary alcohols
			878.82	C–H str.	Aromatic ring vibrations

RCE:* Ricinus communis *extract; WSE: *Withania somnifera* extract; FT-IR: Fourier transform infrared

**Table 3:** Effect of RCE/WSE on hematological parameters of arthritic rats

Treatments	Hematological parameters					
	RBC (10^6^/µL)	Hb (g/dL)	Hct (%)	WBC (10^3^/µL)	Platelets (10^3^/µL)	ESR (mm/h)
**NC**	7.41±0.41	15.38±1.93	40.55±5.97	8.98±1.76	897.83±102.58	8.65±1.09
**AC**	4.29±0.55^*^	9.32±1.94^*^	27.48±2.77^*^	15.06±2.23^*^	1447.60±175.75^*^	13.88±1.12^*^
**MTX**	6.49±1.04^#^	14.25±1.19^#^	34.36±2.19^#^	11.57±2.63^#^	1092.50±74.92^#^	10.33±1.49^#^
**RCE-250**	5.86±0.45^*^^#^	13.53±0.72^#^	32.15±1.65^*^	11.66±1.47^#^	1123.67±93.34^#^	11.10±1.76^#^
**RCE-500**	7.11±0.34^#^	13.99±0.88^#^	35.10±3.63^#^	10.90±2.28^#^	996.70±115.80^#^	9.15±1.51^#^
**WSE-250**	7.34±0.34^#^	13.98±1.06^#^	27.55±2.85^#^^£^	11.96±1.23^#^	1142.17±58.18^*^^#^	11.09±1.75^#^
**WSE-500**	7.74±0.55^b^^£^	15.19±1.24^#^	31.49±3.84^#^	11.35±2.04^#^	1085.70±110.14^#^	9.61±0.89^#^

Values are expressed as mean±SD (n=6). Results were statistically analyzed by one-way ANOVA and Tukey’s post-hoc test. *P<0.01, #*P*<0.05, and £*P*<0.05 difference from NC, AC, and MTX, respectively. Here, NC: normal control; AC: arthritic control; MTX: methotrexate; RCE: *Ricinus communis* extract; WSE: *Withania somnifera* extract

**Table 4:** Effect of RCE/WSE on serum inflammatory, hepatic, and renal parameters of arthritic rats

Treatments	Biochemical parameters						
	CRP (mg/L)	RF (IU/L)	ALP (U/L)	ALT (U/L)	AST (U/L)	Creatinine (mg/dL)	Blood Urea (mg/dL)
**NC**	1.48±0.26	2.98±1.58	95.33±17.76	46.67±5.89	55.33±10.86	0.79±0.23	33.67±6.31
**AC**	3.57±0.88^*^	30.73±3.12^*^	283.00±12.55^*^	160.65±7.19^*^	130.00±6.57^*^	0.74±0.20^ns^	35.62±5.20^ns^
**MTX**	2.39±0.42^#^	16.15±2.69^*^^#^	174.83±12.84^*^^#^	96.67±12.89^*^^#^	96.65±7.98^*^^#^	0.61±0.15^ns^	34.83±6.27^ns^
**RCE-250**	2.83±0.56^*^	16.66±3.41^*^^#^	185.33±15.24^*^^#^	110.50±5.89^*^^#^	111.33±6.59^*^^#^	0.55±0.11^ns^	36.17±6.74^ns^
**RCE-500**	1.74±0.55^#^	10.90±2.28^*^^#^^£^	167.83±10.34^*^^#^	91.25±7.04^*^^#^	90.00±4.86^*^^#^	0.67±0.16^ns^	35.06±5.66^ns^
**WSE-250**	2.49±0.56^#^	16.91±2.72^*^^#^	198.83±16.30^*^^#^	114.83±8.77^*^^#^^£^	115.33±9.46^*£^	0.65±0.16^ns^	36.33±3.56^ns^
**WSE-500**	1.89±0.45^#^	13.24±2.30^*^^#^	175.33±14.24^*^^#^	96.48±4.27^*^^#^	105.33±13.34^*^^#^	0.68±0.18^ns^	33.83±4.75^ns^

Values are expressed as mean±SD (n=6). Results were statistically analyzed by one-way ANOVA, and Tukey’s *post-hoc* test. *P<0.01, #*P*<0.05, and £*P*<0.05 difference from NC, AC, and MTX, respectively. Here, NC: normal control; AC: arthritic control; MTX: methotrexate; RCE: *Ricinus communis* extract; WSE: *Withania somnifera* extract

## Discussion

RA is an autoimmune systemic inflammatory disorder characterized by a specific pattern of bone and joint damage linked to complex disease pathologies (1). The CFA-induced arthritic model has similarities in pathology with human RA, therefore widely employed in preclinical studies using animal models. CFA-induced arthritis comprises two phases; the first acute phase lasting for 10 days accompanying immune cell induced prostaglandins, serotonin, and histamine upsurge, and the following second chronic phase manifested as immune cell infiltration, synovitis, bone erosion, and hypertrophy corresponds to pro-/anti-inflammatory homeostatic disruption (40). 

The present study states the effectiveness of RCE and WSE in adjuvant-induced arthritic Wistar rats. The paw inflammation rate versus time in CFA-induced arthritis comprised initial induction phase without synovitis evidence, early synovitis, and progressive joint damaging late synovitis phase (31). In the present study, RCE and WSE effectively reduced paw inflammation and arthritic score in arthritic rats from day 12 to 24. Weight loss is directly linked to the degree of joint inflammation severity assuming the weight loss might be linked to disease-associated stress, hyper-algesia, reduction in food intake, and malabsorption from the intestine, alteration in lipids and protein metabolism, and muscle proteolysis (31, 41). A significant body weight loss was noticed in the arthritic group that was restored along with a reduction in paw swelling by treatment with both plant extracts. The hyperactivity of the immune system is recognized by splenitis, splenomegaly, and lymphadenopathy (42). The present study revealed suppression of hyper-functioning of immune organs in both extracts treated arthritic rats that might be considered due to the presence of bioactive phytochemicals in plants (43). 

Anemia is one of the prominent clinical features of RA that is significantly restored with both extracts, however, WSE showed higher effects than RCE in contrast to arthritic rats (44)*. *The elevation in serum ALP usually correlates to liver injury and bone erosion. The elevated ALP level also depicted localized bone damage and periarticular osteoporosis (45). The present study indicated restoration of the ALP level in arthritic rats with treatment by both extracts. Histological studies supported the apparent healing of inflamed joints of extracts treated groups. High levels of serum CRP and RF represent active systemic inflammation which indicated RA progression. Significant restoration of CRP and RF levels was observed in extracts treated groups, as compared with the arthritic group. CRP production is aggravated with up-regulation of TNF-α and IL-6 relative expression (46). Results of the present study depicted that RCE and WSE were responsible for reduction in systemic inflammation *via* down-regulation of TNF-α and IL-6 mediated lowering of CRP and RF.

Oxidative stress is assumed to be an underlying pivotal mechanism associated with chronic inflammation in RA (47). CAT and SOD enzymes are an important first-line defense mechanism against free radical species. In this study, depletion of CAT and SOD activities was noticed in liver tissue of arthritic control and both extracts significantly restored CAT and SOD activity. MDA in liver tissue markedly elevated in CFA-induced arthritic rats which was significantly reduced in extracts treated groups. CFA is considered to provoke inflammatory cytokines and ROS that further exacerbate arthritis by triggering immune cells to release enzymes and inflammatory cytokines (46). Thus, reduction in oxidative stress by RCE and WSE might be linked to down-regulation of IL-1β, IL-6, and TNF-α in RA. 

An upsurge of inflammatory cytokines including IL-1β, IL-6, IL-17a, and TNF-α from monocytes and macrophages is evidenced in CFA-induced arthritic rats. An increase in TNF-α relative expression aggravates the discharge of inflammatory mediators that promote immune cell infiltration and edema in inflamed joints (48). Therefore, it necessitates reduction of TNF-α to avoid cartilage and bone damage in arthritis (49). The present study presented a significant reduction in relative gene expression of IL- 1β, IL-6, IL-17a, and TNF-α by both extracts. Moreover, down-regulation of IL-4 and INF-γ anti-inflammatory cytokines markedly attenuated in RCE and WSE treated arthritic rats. OPG and RANKL expressed by osteoblasts play an important role in bone metabolism. RANKL mediates bone resorption by combining with its receptors on osteoclasts. OPG suppresses bone resorption by preventing RANKL binding to its receptors (50). Results of the present study have shown down-regulation of RANKL and up-regulation of OPG in MTX and extracts treated arthritic rats. 

Histopathological findings showed synovial defects including infiltration of inflammatory cells, pannus formation, and bone erosion in the ankle joint of arthritic rats. In contrast, RCE and WSE treatments resulted in therapeutic improvement in articular damage and inflammatory responses in histological analysis. Gross and radiographic observations of ankle joint and soft tissue depicted that both plants at 500 mg/kg markedly attenuated structural alterations and acquired an anti-arthritic effect which might be due to their phytochemical contents. In addition, liver histology of arthritic rats demonstrated significant hepatocyte necrosis, vacuolization, and mononuclear cell infiltration which were markedly reduced in RCE and WSE (500 mg/kg) treated arthritic rats. 

MTX decreased IL-1β, IL-6, IL-17a, TNF-α, and RANKL relative expression while increasing IL-4, INF-γ, and OPG relative expression in arthritic rats along with restoring body weight and reducing paw inflammation. The findings of the present study supported by previous studies on MTX and plant extract revealed ameliorative effects in arthritis (51). Therefore, it could be speculated that a reduction in oxidative stress by RCE and WSE is one of the main mechanisms for modulation of inflammatory mediators in RA. 

Phytochemical analysis showed the presence of flavonoids and polyphenols such as gallic acid, quercetin, benzoic acid, caffeic acid, ferulic acid, cinnamic acid, coumaric acid, chlorogenic acid, *m-*coumaric acid, *p-*coumaric acid, syringic acid, and vanillic acid. It has previously been demonstrated that polyphenols exhibit significant anti-oxidant activity due to their high hydroxyl group content and scavenge ROS (40). Studies reported that flavonoids and polyphenols present in RCE and WSE are responsible for their potential anti-oxidant activity (18, 23). 

## Conclusion

It is evident from the present study that RCE and WSE have marked anti-arthritic potentials, and hence could be implicated in RA management. The anti-arthritic activity may be linked to the bioactive phytoconstituents. Therefore, *R. communis *leaves and *W. somnifera *roots can be used as herbal remedies for RA and inflammatory diseases. 
